# Impact of Different Types of Physical Activity in Green Urban Space on Adult Health and Behaviors: A Systematic Review

**DOI:** 10.3390/ejihpe11010020

**Published:** 2021-03-04

**Authors:** Alessia Grigoletto, Mario Mauro, Pasqualino Maietta Latessa, Vincenzo Iannuzzi, Davide Gori, Francesco Campa, Gianpiero Greco, Stefania Toselli

**Affiliations:** 1 Department of Biomedical and Neuromotor Sciences, University of Bologna, 40126 Bologna, Italy; alessia.grigoletto2@unibo.it (A.G.); davide.gori4@unibo.it (D.G.); stefania.toselli@unibo.it (S.T.); 2 Department of Basic Medical Sciences, Neuroscience and Sense Organs, University of Study of Bari, 70121 Bari, Italy; mario.mauro.194@gmail.com; 3 Department of Life Quality Studies, University of Bologna, 47921 Rimini, Italy; pasqualino.maietta@unibo.it (P.M.L.); francesco.campa3@unibo.it (F.C.); 4 Department of Statistical Sciences, University of Bologna, 40126 Bologna, Italy; vincenzo.iannuzzi2@unibo.it

**Keywords:** exercise, health, natural environment, training

## Abstract

This systematic review aimed to investigate the type of physical activity carried out in green urban spaces by the adult population and to value its impact on the population’s health. Additionally, another purpose was to examine if the presence of outdoor gyms in green urban spaces can promote participation in physical activity among adults. Searches of electronic databases, with no time restrictions and up to June 2020, resulted in 10 studies meeting the inclusion criteria. A quantitative assessment is reported as effect size. Many people practiced walking activity as a workout, which showed improvements in health. Walking is the most popular type of training due to its easy accessibility and it not requiring equipment or special skills. Outdoor fitness equipment has been installed in an increasing number of parks and has become very popular worldwide. Further, outdoor fitness equipment provides free access to fitness training and seems to promote physical activity in healthy adults. However, other studies about outdoor fitness equipment efficiency are needed. People living near to equipped areas are more likely to perform outdoor fitness than those who live further away. The most common training programs performed in green urban spaces included exercises with free and easy access, able to promote physical health and perception.

## 1. Introduction

By 2030, three out of every five people of the worldwide population will live in an urban area [1]. Therefore, one of the most important challenges for the future will be to create people-friendly cities and safeguarding green spaces will be fundamental in achieving this. Green spaces are defined as open spaces of ground, partially or completely covered by vegetation, including parks and gardens; they can be considered important because the characteristics of the environment in which people live are linked to the quality of their health, both physical and mental [2]. There has been an increase in the literature focused on the importance of green urban spaces and several studies have highlighted a relationship between exposure to the natural environment and better health perception [2], but the mechanisms that explain this relationship are not so clear [3,4]. Different studies proposed different types of mechanics that could be summarized in the following ways: (1) restoration theory, in which the intrinsic quality of natural outdoor environments influences the health perception and well-being from seeing or watching a green space [5,6,7]; (2) biodiversity increase, the link between green space and a healthy environment, which is influenced by the immune response, lower temperature and lower air and noise pollution [8,9,10,11,12,13,14,15,16]; (3) opportunity to perform physical activity (PA) [5,6,16,17]; (4) enhancement of social interaction [17,18,19,20]. 

One of the key factors in improving global public health is PA because the beneficial effects of a physically active lifestyle on various health outcomes are well established, with strong evidence of risk reduction for chronic diseases and cognitive and functional decline and improvement in mental health [21,22]. Moderate physical activity intensity compared to a total sedentary behavior can reduce the relative risk of mortality [23]. Despite this, it is estimated that 3.3 million people die annually because of physical inactivity, making it the fourth leading cause of non-communicable death worldwide [24], and a significant proportion of the adult population remains inactive [25]. To help in reducing the burden of chronic diseases and morbidity due to an inactive lifestyle, effective interventions are needed in increasing PA in the general population [26]. Green urban space could be a solution for this problem because the exposure to a natural environment is also linked with triggering a higher amount of PA carried out by residents, and a lower mortality ratio [2,6,17,27]. Experimental research suggested that performing PA in nature may have additional benefits in comparison with a period of PA in an indoor environment [28]. Further, as already reported, exposure to nature could improve people’s health and well-being, by providing restoration from stress and mental fatigue [29]. Green urban space could reach a variety of people due to it being freely accessible, and it could help PA levels in people who do not usually perform exercise [30,31,32,33]. Due to the growth of urban space, there has been the need to upgrade open urban spaces or built environments to promote PA [34,35,36,37,38,39,40,41,42]. Existing literature on the physical activity performed in green urban spaces showed high levels of heterogeneity in samples, intervention characteristics and investigated outcomes. Specifically, there has been a lot of different types of intervention in green urban space as resistance training using outdoor gyms and aerobic physical activity (walking, running, biking), but it is not known if they could have different effects on health [43]. 

This systematic review has the objective to outline a picture of different types of physical activity proposed in green urban spaces. Further, this review focuses on different outdoor trainings to understand their impact on the population’s health. Finally, we wanted to clarify whether the presence of outdoor gyms in green urban spaces can promote participation in physical activity in adults. 

## 2. Materials and Method

### 2.1. Search Strategy 

The Preferred Reporting Items for Systematic Reviews and Meta-analysis (PRISMA) guidelines were followed to conduct this systematic review [44]. Databases used included: Cochrane Library, Med-Line, SportDiscuss, GreenFile, Health Administration Database, The UK and Ireland Database and Psycinfo. Search strategies were adapted to the different databases and these keywords and terms were used: “outdoor exercise (exercises)” OR “outdoor fitness” OR “outdoor physical activity” OR “green urban space exercise (exercises)” OR “green urban space physical activity” OR “outdoor training” OR “outdoor circuit training” OR “outdoor resistance training” OR “outdoor high intensity training” OR “park exercise” OR “park training” AND “adult”. Terms were searched as titles and keywords. 

### 2.2. Eligibility Criteria

Table 1 shows inclusion and exclusion criteria. Population, Interventions, Comparators, Outcomes, Study design and Timing (PICOST) of interest were defined and different electronic databases were used to search the keywords with no time restrictions up to 10 June 2020 (T) [45]. The Population (P) was adults aged between eighteen and eighty years, and with no chronic diseases or health problems. Children and teenagers were excluded because they are a specific sector of the population with specific needs. Interventions (I) of interest were physical activity carried out in green urban spaces or parks to evaluate how PA could affect adult health; gym equipment installation to evaluate its impact on adult behavior. Physical activity indoors or in different natural environments such as beaches or blue areas was excluded. Comparators (C) were the control group (if presented); baseline observation; and park with no gym equipment. If participants in the studies received different treatments from PA, they were excluded. Outcomes (O) were impact of PA in green urban spaces and/or PA and health indicators and behavior characteristics of park users. Only observational or experimental studies, written in English, with original primary data, were selected (S). Papers with no study protocol or other papers without original data were analyzed.

### 2.3. Article Information

After quality assessment, a double-blind extraction of data was performed. This included: author, country, study design, population, type of interventions, intensity and frequency of the intervention, outcomes, number of experimental and/or control groups, results and studies’ stratification for the different types of interventions. 

### 2.4. Effect Size and Treatment Effect

Two independent reviewers extracted data available in the studies (MM, AG). The statistical analysis was assessed to quantify the effect size (ES) or treatment effect (TE) for each study. The principal summary measures were expressed as standardized differences in means (Cohen’s d) of CRF and *p*-values to quantify the statistical significance of the evidence. It is assumed that an effect size *d* ≥ 0.80 represents a large effect, 0.50 ≤ *d* < 0.80 medium and *d* < 0.50 small [46]. Further, Hedge’s g estimator was used to calculate unbiased *d* values, using the J correction factor. When proportions, correlation coefficients and odds ratios were found, we appropriately converted them among ES [47]. Finally, we calculated the statistical test value (Z or Student’s *t*) where the *p*-value was not shown.

## 3. Results

**Figure 1:**
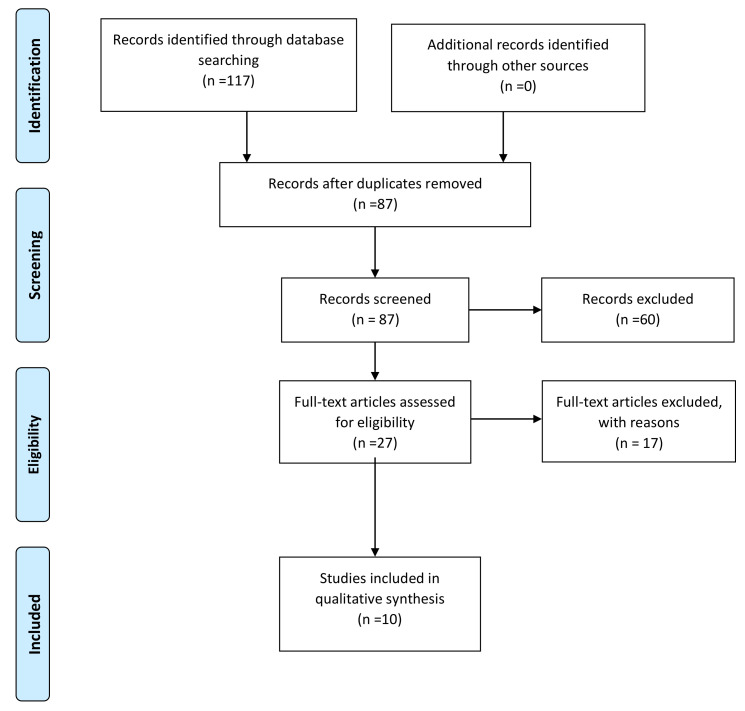
PRISMA flowchart.

One hundred and seventeen articles were retrieved from the browsed databases. Thirty studies were excluded because they were duplicated, and 60 articles were excluded following abstract and/or title review. Twenty-seven studies were classified as pertinent, but 17 were subsequently excluded after detailed full-text reading. In the end, the articles included in the systematic review totaled 10 which fully met the eligibility criteria (Figure 1).

### 3.1. Participant Characteristics

Table 2 shows the participants’ characteristics. Their geographic origins were Australia (three articles, 30%), Taiwan and Korea (two articles, 20%), the USA (two articles, 20%), Ukraine, Sweden and the UK (one article for each, for a total of three articles, 30%). 

The sample size varied from 6 participants to 203883 participants [48,49,50,51,52,53,54,55,56,57]. Two studies included different age classes of participants: 2–4, 5–18 or more than 18 years [52]; children, adult and senior [50]. Other studies explained the weighted average age of the samples. A different distribution of gender resulted from them; only one study reported a whole male sample [53].

### 3.2. Impact of Outdoor Fitness on Participants’ Health 

Table 3 reports the main characteristics and results of the studies which analyzed the outdoor fitness effect on participants’ health. The articles presented a big heterogeneity in the study design, number of participants, time of experiment, type of treatment, measurements performed and statistical analysis. Nevertheless, this highlighted the efficacy of outdoor PA on participants’ heath. The study designs were randomized control trials (RCTs) or control trials (CTs) with no randomization. Three studies of six reported one experimental group and one control group [54,55,56]; one of these reported two experimental groups [56]. One study reported a cross-sectional analysis [51]. Two studies compared differences in time, between pre- and post-evaluation [48,53]. The duration of the studies varied from 6 weeks to 6 months [54,56]. Three studies had a walking program as the treatment [51,53,55] and three treated participants with different outdoor activities [48,54,56]. 

The randomized control trials (RCTs) [55,56] evaluated two different treatments (walking program and outdoor activity) on several measurements (fatigue perception; upper body muscular strength/endurance and physical function). Both the studies showed improvements on EGs compared to CG (with no PA intervention). Wu et al. [55] reported a significant statistical difference in overall fatigue among the two groups (*p* < 0.001; *g* = 1.313). Further, the brisk walking intervention had a positive effect on motivation for the EG (*p* < 0.05), improved concentration for EG (*p* < 0.05) and did not affect the reduction in activity. Kim et al. [56] analyzed two experimental groups (resistance exercise, n = 12; combined resistance and aerobic exercise, n = 13) and one control with no exercise (n = 10). They used the American College of Sports Medicine (ACSM) guidelines for the elderly population to select the frequency, intensity and duration of PA. Both experimental groups (EGs) showed significative improvements in upper body muscular strength/endurance, measured by a push-up test (*p* = 0.017; *g* = 0.385), and physical function, measured by a six-min walking test (*p* = 0.003; *g* = 0.395), compared with the control group. However, the small sample size was a research limitation. 

Two control trials (CTs) analyzed the efficacy of a group-based walking program in an outdoor environment, in which PA intensity varied from light to moderate to vigorous (MVPA) [51,53]. The treatment proposed by Schoffman and colleagues [51] improved participants’ self-efficacy, increased the percentage of weekly MVPA and showed a positive correlation between walking activity and perceived happiness. In addition, Marselle et al. [53] found that perceived restorativeness and perceived naturalness interacted to enhance the positive effect following an outdoor group walk (both *p* < 0.001) and that the intensity of the walking program could change the perceived well-being of participants (*p* < 0.0001). 

Two CTs evaluated how different outdoor activities (combined and resistance training) affected physiological measurements [48,54]. Both the studies showed improvements in physical fitness after the treatments. Apaychev et al. [54] compared the experimental group (EG, n = 20), who performed a combined outdoor training program (resistance and aerobic exercise), with the control group (CG, n = 20), who performed a combined indoor training program (resistance and aerobic exercise). They also reported significant changes (*p* < 0.05) in the indices of health state, activity and mood of EG men. Differently, Johnson et al. [48] analyzed six participants pre- and post-treatment (paired group). The researchers designed a resistance training program in which participants could select one of two training sessions, either 20:10 s or 40:20 s (work:rest). The overall results showed an average increase from baseline to the post-measures in strength, time to exhaustion (cardiovascular fitness; *p* < 0.05; *g* = 0.76) and the number of steps (*p* < 0.01; *g* = 0.02). In addition, the small sample size was a big limitation.

### 3.3. Impact of Green Spaces on Physical Activity Behaviors 

Table 4 shows the factors which can influence the use of green spaces. Four observational studies analyzed different sample sizes, from 358 to 203,883 participants [49,50], which lasted differently (12 up to 16 months) [52,57]. 

Three studies analyzed the impact of gym installations on park usage [50,52,57]. Two of these analyzed the differences in park user customs, before and after the outdoor gym equipment installation, and showed an increase in park usage [50,52]. Further, a higher MVPA frequency was found after OFE installation [50]. The other one assessed a comparison between parks with equipment and parks with no equipment [57]. The authors showed a significant difference in the proportion of PA practiced in the two different contexts. The presence of outdoor fitness equipment (OFE) positively affected the promotion of physical activity. 

**Figure 2:**
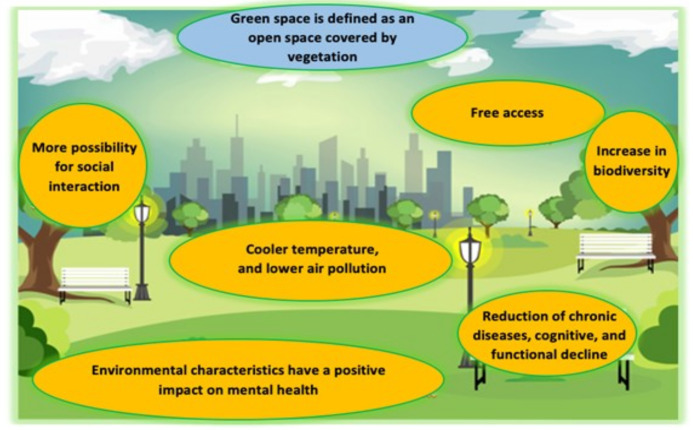
What is a green space?

Astell-Burt and colleagues studied the impact of distance from green spaces, in order to understand whether people who lived nearer green spaces performed more PA than people who lived further away [49]. They reported the time spent walking and moderate to vigorous physical activity. The researchers found significant differences between participants who lived with the availability of 0–20% and those who had +80% availability in walking and MVPA. Greener neighborhood environments positively affected the frequency of participation in walking activity and MVPA. Figure 2 shows what a green space means.

### 3.4. Summary Statistics 

**Figure 3:**
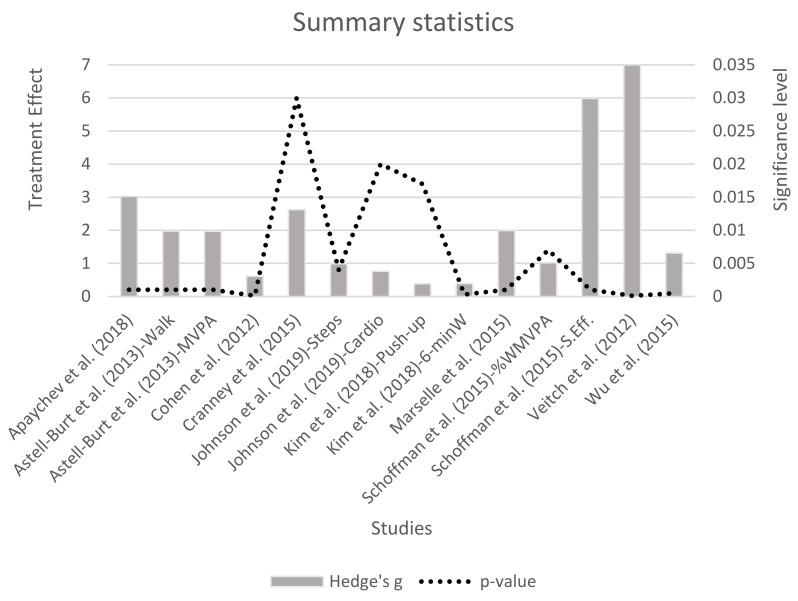
Summary statistics.

Figure 3 shows summary statistics with the treatment effect (g) and *p*-value for each study analyzed. Four studies reported two different data: Astell-Burt et al. reported outcomes for a walk and MVPA [49], Johnson et al. reported outcomes for the number of steps and cardiovascular fitness [48], Kim et al. reported outcomes for the number of push-ups and a 6-minute walk [56] and Shoffman et al. reported outcomes for the percentage of weekly MVPA and self-efficacy [51]. Veitch and colleagues observed the highest treatment effect (difference in proportion, *g* > 7) [52]. Eight studies reported a large ES (*d* > 0.80); only Cohen and colleagues showed a moderate TE (0.50 < *d* < 0.80), and only Kim et al. showed a small ES (*d* < 0.50). 

## 4. Discussion

This systematic review aimed to outline a picture of the different types of physical activity proposed in urban green spaces. Further, this review focused on different outdoor trainings to understand their impact on the population’s health. Finally, we wanted to clarify whether the presence of outdoor gyms in green urban spaces could promote participation in physical activity in adults. Existing literature on PA performed in green urban spaces shows high levels of heterogeneity for samples, intervention characteristics and investigated outcomes; specifically, there are a lot of different types of interventions proposed, but it is not clear if they have similar effects on health. This review aims to fill this gap and so, unlike the previous systematic review, it is focused on how different types of interventions affected participants’ health. 

The systematic research of the literature found 10 studies, 6 of which analyzed the impact of different outdoor PA on participants’ health and 4 of which observed the effect of green spaces on participants’ behaviors.

In this review, several types of training were reported, and among all those considered, walking training, resistance exercise and combined exercise (resistance and aerobic) showed the best results on participants’ health.

Many epidemiological studies highlighted the health benefits of walking. PA, including walking, has a substantial role in the management of coronary heart disease, hypertension, type 2 diabetes, obesity, elevated cholesterol, osteoporosis, osteoarthritis, obstructive pulmonary disease and several other conditions, including depression and anxiety disorders, dementia, pain, congestive heart failure, syncope and stroke [58]. Walking as a healthy form of PA began to receive attention in the 1990s because of new recommendations by the American College of Sports Medicine that emphasized the importance of a healthy lifestyle and MVPA. Then, also the World Health Organization (WHO) created its guidelines on PA and sedentary behavior, in which the last update was in 2020. The WHO Guidelines highlighted the health benefits of a greater amount of PA, including light-intensity PA and the importance of breaking up sedentary time with light-intensity activity [59]. Lee et al. [60] observed an inverse relationship between overall walking and the risk of developing coronary heart disease, in women. Walking is the easiest way to remain active and the most popular, so it is one of the major focuses in the PA initiative. It is the most reported activity in adults who meet physical recommendations [61]. It is probably because of its accessibility. Walking is a universal form of PA that is appropriate regardless of sex, ethnic group, age, education or income level. Walking does not require expensive equipment, special skill or special facilities. Walking is also important for older adults. Walking outdoors at least once a week has been associated with achieving more time spent in MVPA than walking indoors [58] and it also provides a means to participate in meaningful activities, such as shopping or leisure activities (e.g., visiting friends or pleasure walking). Limited walking is also effective in preventing falls and fall injuries in older adults [62]. A meta-analysis of four studies that included walking reported a 44% reduction in fall injuries in the intervention group [63]. Therefore, the costs of medical care are substantially lower in physically active adults [64], and walking has the potential to reduce medical expenditure, particularly among older adults where the prevalence of the chronic disease is higher [65]. Wu and colleagues demonstrated that a walking program activity could improve perceived fatigue [66]. Further, a walking program reported an improvement in the happiness of the participants [57]. 

Some studies reported the beneficial effect of RE and CO outdoor PA on adult health. Improvements in cardiovascular fitness, strength and endurance were shown [52,67]. However, many articles are needed to confirm the good impact of resistance and combined outdoor PA on adult wellness. 

Our results show that the presence of many green spaces could increase walking and MVPA [54]. Moreover, the presence of park equipment can favor walking activity [68]. 

Many studies reported that OFE has become very popular worldwide in numerous green spaces and built-up environments [69,70,71,72,73,74,75,76]. According to an investigation by Chow [77], OFE was installed in more than half of the parks in cities in Taipei and a growing number are being added to parks in the United States [75], South America [76], Australia [77] and some European countries, such as Spain [73] and Portugal [72]. OFE could be used by everyone because it provides free access to fitness training for the community and also enables a different kind of training (e.g., resistance or circuit training) [73,74,75]. The installation of OFE in green urban spaces offers many benefits, including increasing engagement in PA [76,78], improving the perception of security, adding pleasant contributions to the city’s landscape [78,79] and encouraging social interaction [76,78]. Some studies found different benefits directly associated with OFE, such as improved cardiorespiratory fitness, muscle strength, balance and flexibility [52,63], but the studies also reported limited and mixed results, so the effects of OFE have not yet been fully explored. The presence of OFE in green urban spaces could attract new visitors and increase the overall number of park visits [80]. Our review agrees with the above results.

However, this is in contrast with what Cranney et al. found in their study. In the article, they reported a significant short-term increase in MVPA among the overall park users, especially after the OFE installation, but they also suggested it was maybe due to a seasonal effect because the installation occurred in summer, and at the post-installation control, in the autumn, there was not an increase in park use. The authors suggested that the OFE may not attract new park users but may provide existing park users with more opportunities for active recreation [81]. According to this result, Chow et al. [82] observed that most users interacted with fewer than three OFE stations (out of a total of six) available in the park and each OFE user operated one device for less than five minutes with a total time using all equipment of fewer than nine minutes. This is coherent with an observational study, which reported that many users used OFE only for a very short period, which could be insufficient to produce substantial health benefits [82]. Earlier studies [52] claimed that MVPA was achieved using OFE, but Chow et al. [78] investigated the energy expenditure and level of intensity during OFE use and the result was that the use of OFE appeared to be less intense compared with the use of conventional resistance training machines in indoor gyms. This result does not represent all kinds of OFE because there are different manufacturers that design and produce OFE, with differences in size, shape, materials or smoothness of operation. The lack of energy expenditure could be compensated by the natural environment, which brings additional benefits in comparison with PA in an indoor environment and can improve people’s health and well-being by providing restoration from stress and mental fatigue [28,29]. People who constantly perform PA could also see more positive effects due to a different setting. 

Heterogeneous studies have indicated that OFE could pose many safety problems because of a lack of surveillance and inadequate usage instructions [78], and they also reported many OFE accidents due to users operating equipment incorrectly [79,80]. A survey study indicated that many users mimic how others use equipment because no information session was conducted after installing the OFE and many instructions were absent [78]. Therefore, an important development for the future and the increase in OFE use could be that manufacturers provide clear equipment operation guides (or demonstration videos) on the correct use of their equipment and warning messages regarding risky behaviors. Manufacturers should also design OFE with suitable angle ranges or fixed operating positions. The government or the local authorities that authorized the OFE installation may allow instructional sessions in which professional trainers can explain how to safely use the OFE to meet the individual’s capability and fitness level [80,81]. 

### 4.1. Limitations 

The biggest limitation of this study is linked to a lack of findings/definitive results and articles in the literature. It was possible to include in this systematic review only 10 articles. The literature about the general importance of green urban spaces is growing, but studies about constructing interventions in green urban spaces for the general population are not growing at the same speed. There are a lot of studies about the beneficial effects of natural environments for children, teenagers and the elderly population to promote an active lifestyle or a healthy elderly age. However, the growing literature is not connecting green urban spaces and rehabilitation interventions. This can be of paramount importance to create specific protocols for people undergoing rehabilitation programs for different health problems, such as cardiovascular diseases. Green urban spaces could also be a good support or an alternative for this kind of rehabilitation. In green urban spaces, there are also physical activity protocols for people with chronic diseases, such as diabetes. There is no doubt that these kinds of problems and protocols are very important to promote public health, but there is a lack of literature on physical activity for the general population, such as people between eighteen and seventy-five years old, without health problems. This is a big part of the population that is very little considered in the literature.

For these reasons, the sample of articles that met the inclusion criteria was very limited. It was not a problem of quantity, but rather quality, because a major part of the articles retrieved provided an “intermediate”-quality evaluation, with different problems and lack of information. Furthermore, the articles were very heterogenous, and they had a different approach (i.e., the simple observational approach before/after, without randomization), so it was difficult to obtain strong and definitive evidence and conclusions. 

### 4.2. Future Implication 

The importance of PA and its positive effects are well known, but even if there is increasing literature also about the efficacy of PA performed in a natural environment, the evidence is not so clear. There are very few studies that compared the effects of different types of activity performed in green urban spaces, and there are very limited recommendations about the kind of training, the intensity of exercises or the time to spend performing PA in green urban spaces. Therefore, more specific studies are needed. 

## 5. Conclusions

Outdoor activity may improve adult health. The most popular activity performed in green urban spaces resulted as walking and the use of OFE. Walking is an easy activity, performed every day by people to move, so it does not require special skills or particular training to be performed. Walking is also an activity with no cost because people can perform it in a city’s park or anywhere they want for free, and they do not have to pay a subscription. Moreover, people can walk when it is best for them because there usually is no time restriction for park use. This kind of activity does not require particular equipment, people can wear what they prefer and walking can be considered as a workout or a recreational activity, such as walking the dog, but in any case, it is a good way to maintain a healthy lifestyle. OFE installations could promote physical activity, but other studies to better understand their effects on health are needed.

## 6. Future Research Directions

Future studies should propose different kinds of PA in addition to walking and the use of OFE, in order to have the possibility to understand what the best kind of training in an outdoor environment is. Moreover, it will be important to have other studies with a clear explanation of the protocols used and proposed.

## Figures and Tables

**Table 1:** PICOST eligibility criteria.

Parameter	Inclusion Criteria	Exclusion Criteria
Population	Healthy adults (18 ≤ age ≤ 80)	People < 18 or >80
		Unhealthy people
Intervention	PA in green urban spaces and gym equipment installation	PE indoors or in different natural environments such as beaches or blue areas
Comparator	Control group (if presented); baseline observation; park with no gym equipment	Participants receiving different protocol from PA
Outcome	Impact of PE in green urban spaces and/or PA and health indicators and behavior characteristics of park users	No information about PA
Study design	Observational or experimental with original primary data	Study protocols or other papers without original data
	English language	Not in English language
Timing	No time restrictions until 10 June 2020	After 10 June 2020

Abbreviations: OFE = outdoor fitness equipment; PA = physical activity; PE = physical exercise; PSY = psychological.

**Table 2:** Participant characteristics.

Study	Country	Participants	Age (yrs.)	Gender
Veitch et al. (2012)	Australia	1309	≥18	M and F
Cohen et al. (2012)	USA	2636	40 ± 12.5 *	F = 61%; M = 39%
Wu et al. (2015)	Taiwan	86	31.28 ± 4.93 *	F = 50%; M = 50%
Johnson et al. (2019)	Sweden	6	41.2 ± 6.5 *	F = 33%; M = 67%
Apaychev et al. (2018)	Ukraine	60	40–50	M
Schoffman et al. (2015)	USA	295	49.4 ± 13.3 *	F = 86%; M = 14%
Marselle et al. (2015)	UK	127	55–74	F = 55.5%; M = 44.5%
Astell-Burt et al. (2013)	Australia	203883	61.5 *	F = 53.2%; M = 46.8%
Cranney et al. (2015)	Australia	796	≥18	F = 47.6%; M = 52.4%
Kim et al. (2018)	Korea	35	73.2 ± 4.95	F = 91.5%; M = 8.5%

* Weighted average of age ± SD (if explicated); M = male; F = female.

**Table 3:** Impact of outdoor fitness.

Study	Design	EG	CG	Other Group	Treatment	Duration	Cohen’s d	Hedge’s g	*p*-Value	Measurements	Analysis
Wu et al. (2015)	RCT	41	45	no	Walking program	8 weeks	1.325	1.313	0.0005	Overall fatigue	Stand. Mean diff.
Schoffman et al. (2015)	CT	Cross-sectional			Walking program	6 months	1.036	1.02	0.007	%Weekly MVPA	Correlation coefficient
		194		no			6.08	5.99	>0.001	Self-efficacy	
Marselle et al. (2015)	CT	Pre	Post		Walking program	13 weeks	2	1.99	>0.001	Walk happiness	Correlation coefficient
		127	127	no							
Apaychev et al. (2018)	CT	EC	CG		Outdoor activity	6 months	4.02	3.03	>0.001	Motor activity	Stand. Mean diff.
		20	20	no							
Johnson et al. (2019)	CT	Pre	Post		Outdoor activity	10 weeks	1.08	0.99	0.004	Number of steps	Stand. Mean diff.
		6	6	no			0.83	0.76	0.02	Cardiovascular fitness	
Kim et al. (2018)	RCT	EG1	CG	EG2	Outdoor activity	6 weeks	0.4	0.385	0.017	Number of push-ups	Stand. Mean diff.
		12	10	13			0.41	0.395	0.0003	6-min walk	

Abbreviations: RCT = randomized control trial; CT = control trial; EG = experimental group; CG = control group; MVPA = moderate to vigorous physical activity; Stand. Mean diff. = standardized mean difference.

**Table 4:** Impact of factors that can influence the use of green spaces.

Study	Design	Participants	Exercise Type	Duration	Cohen’s d	Hedge’s g	*p*-Value	Outcome	Measurements	Analysis
Astell-Burt et al. (2013)	OBS	203883	Walking and MVPA	not specified	1.98	1.98	>0.001	Impact of distance on park usage	Walking near green space	Odds ratio difference
					1.97	1.97	>0.001		MVPA near green space	
Cranney et al. (2015)	OBS	358	Outdoor gym	12 months	2.63	2.63	>0.03	Impact of gym installation on park usage	MVPA frequency pre–post	Proportion difference
Cohen et al. (2012)	OBS	958	Outdoor gym	16 months	0.61	0.61	>0.0001	Exercise in gym park vs. no gym park	Exercise frequency	Proportion difference
Veitch et al. (2012)	OBS	609	Outdoor gym	12 months	<7	<7	>0.0001	Impact of gym installation on park usage	Usage frequency pre–post	Proportion difference

Abbreviations: OBS = observational study; MVPA = moderate to vigorous physical activity.
